# Two novel effectors of trafficking and maturation of the yeast plasma membrane H^+^‐ATPase

**DOI:** 10.1111/tra.12503

**Published:** 2017-08-16

**Authors:** Yosef Geva, Jonathan Crissman, Eric C. Arakel, Natalia Gómez‐Navarro, Silvia G. Chuartzman, Kyle R. Stahmer, Blanche Schwappach, Elizabeth A. Miller, Maya Schuldiner

**Affiliations:** ^1^ Department of Molecular Genetics Weizmann Institute of Science Rehovot Israel; ^2^ Department of Biological Sciences Columbia University New York NY; ^3^ Department of Molecular Biology Universitätsmedizin Göttingen Göttingen Germany; ^4^ MRC Laboratory of Molecular Biology, Cell Biology Division Cambridge UK

**Keywords:** Ydl121c, Exp1, Ykl077w, Psg1, Pma1, COPI, COPII, Lst1, Sec24, Kex2

## Abstract

The endoplasmic reticulum (ER) is the entry site of proteins into the endomembrane system. Proteins exit the ER via coat protein II (COPII) vesicles in a selective manner, mediated either by direct interaction with the COPII coat or aided by cargo receptors. Despite the fundamental role of such receptors in protein sorting, only a few have been identified. To further define the machinery that packages secretory cargo and targets proteins from the ER to Golgi membranes, we used multiple systematic approaches, which revealed 2 uncharacterized proteins that mediate the trafficking and maturation of Pma1, the essential yeast plasma membrane proton ATPase. Ydl121c (Exp1) is an ER protein that binds Pma1, is packaged into COPII vesicles, and whose deletion causes ER retention of Pma1. Ykl077w (Psg1) physically interacts with Exp1 and can be found in the Golgi and coat protein I (COPI) vesicles but does not directly bind Pma1. Loss of Psg1 causes enhanced degradation of Pma1 in the vacuole. Our findings suggest that Exp1 is a Pma1 cargo receptor and that Psg1 aids Pma1 maturation in the Golgi or affects its retrieval. More generally our work shows the utility of high content screens in the identification of novel trafficking components.

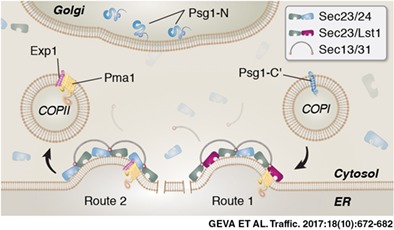

## 1 INTRODUCTION

The endoplasmic reticulum (ER) is the entry site for proteins into the endomembrane system. Once appropriately folded, non‐ER resident proteins are routed to the Golgi apparatus for further maturation and distribution.^1^ Proteins exit the ER via coat protein II (COPII) vesicles. Although some proteins are captured by stochastic sampling of the ER lumen and membrane in a process called bulk flow,^2^, ^3^ more efficient and regulated sorting relies on selective uptake into vesicles. Additional sorting specificity occurs by retrieval of ER residents or immature proteins from the Golgi apparatus to the ER through coat protein I (COPI) vesicles.^4^, ^5^ The general mechanisms of vesicle formation are well characterized, yet the molecular basis that governs specific recognition between the coats, and the diverse range of proteins that must be packaged into vesicles, is yet to be fully described.

The cargo binding subunit in the COPII coat is Sec24. One of the characteristics of Sec24 that enables it to bind many diverse cargoes is the presence of 3 isoforms: Sec24, Iss1 (Sfb2) and Lst1 (Sfb3), each with multiple distinct binding sites for cargo or adaptors.^6^, ^7^, ^8^ Each Sec24 isoform will yield COPII vesicles that differ in their cargo and lipid content.^9^, ^10^ For example, the Lst1 isoform is necessary for efficient traffic of some GPI‐anchored proteins^11^ and Pma1, the plasma membrane H^+^‐ATPase,^12^ 2 classes of proteins that reside in sterol‐ and ceramide‐/sphingolipid‐rich domains.^13^

Importantly, although combinatorial use of Sec24 isoforms increases binding opportunities, this is unlikely to account for the full spectrum of cargo diversity handled by the COPII system. Additional diversity stems from the use of cargo receptors, whose role is to bind cargo and indirectly connect it to a specific binding site of a Sec24 family protein^14^, ^15^, ^16^, ^17^, ^18^, ^19^, ^20^, ^21^. Some cargo receptors further increase the diversity of their cargo capture by binding additional adaptors.^22^, ^23^, ^24^ Finally, additional modes of Sec24 interaction can also increase diversity and specificity: some cargoes are capable of binding 2 separate sites^6^; others bind cooperatively through both direct Sec24 interaction and indirect receptor‐mediated interaction.^8^

Currently, there are around 15 known cargo receptors in the budding yeast, *Saccharomyces cerevisiae*.^5^ Taking into consideration the hundreds of proteins that must be effectively sorted into COPI or COPII vesicles at any given moment, even in the simplest eukaryotic model organism, yeast, the existence of additional cargo receptors and adaptors is postulated. Here, we used mutations in the binding sites of yeast Sec24 to perturb ER to Golgi traffic in the context of more than 374 individually tagged yeast proteins targeted to the secretory pathway. This approach coupled with a high‐content microscopic screen revealed a novel cargo receptor for the essential yeast plasma membrane H^+^‐ATPase, Pma1.

## 2 RESULTS

### 2.1 A high‐content screen uncovers an uncharacterized protein that cycles between the ER and the Golgi apparatus

To better understand the regulation of protein flux through the secretory pathway, we aimed to define new cargo receptors. One of the hallmarks of a cargo receptor is that it dynamically cycles through the ER and Golgi via COPII and COPI vesicles. However, cargo receptors are not usually visualized in all these compartments in parallel but tend to hold a single, typical, steady‐state localization. We hypothesized that by perturbing the ER‐Golgi cycle we could capture cargo receptors in a stereotypical way that would allow us to identify novel candidate receptors. To this end, we visualized a large number of secretory pathway proteins tagged with green fluorescent protein (GFP) on the background of perturbed ER‐Golgi traffic. Specifically we selected 374 strains each expressing a protein that resides in the secretory pathway that is genomically tagged with GFP at its C terminus.^25^ This “secretome library” encompasses proteins localized to either the ER, Golgi apparatus, plasma membrane, vacuolar membrane, vacuolar lumen, COPI or COPII vesicles, peroxisomes, undefined punctate localization and secreted proteins (Table S3, Supporting information).^19^

**Figure 1:**
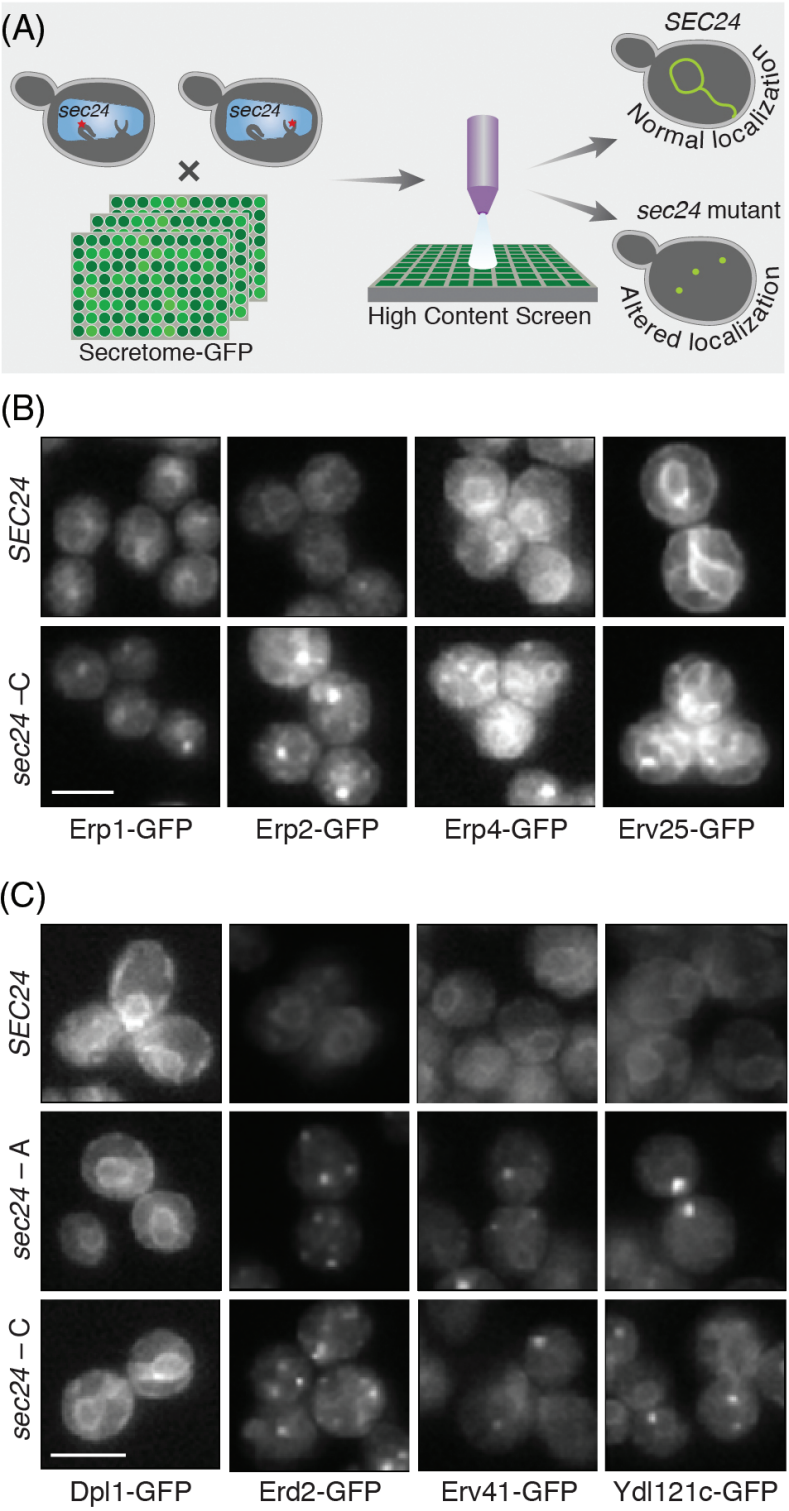
A systematic approach identifies a potential new cargo receptor. A, To uncover new cargo receptors we integrated mutations in *sec24‐A* or *C* cargo‐binding sites into every strain of the yeast secretome‐GFP collection. We then followed the localization of each protein by high‐content screening and manual comparison between the mutated and the control strains. B, The mutation in the *sec24‐C* binding site resulted in localization changes of 4 cargo receptors of the p24 protein family. The cargo receptors alter their localization from ER in the control cells (*SEC24* wild‐type) to punctate localization in the mutant (*sec24‐C*). C, Most proteins did not change localization as a result of mutations in *sec24‐A* or *C* cargo‐binding sites compared with the control (*sec24‐A/C*/wild‐type, respectively). Dpl1 is an example for such a protein. Three proteins changed their localization from ER to punctate pattern in both mutants: Erd2, Erv41 and Ydl121c. Bar = 5 µm

To perturb traffic we chose to use mutations in the cargo binding sites of Sec24 as it was already shown that a mutation in the Sec24‐A binding site perturbs ER‐Golgi vesicular traffic.^26^ Moreover, the 2 best‐characterized cargo of the A‐ and C‐sites are SNARE proteins that act in anterograde (Sed5) and retrograde (Sec22) traffic, suggesting that bidirectional traffic between the ER and Golgi would be perturbed in these mutants. In contrast, many cargo that engage the B‐site are proteins that move forward in the secretory pathway. Thus we focused on A‐ and C‐site mutations that should more specifically perturb ER‐Golgi vesicular traffic.^6^, ^7^ We used automated mating techniques to integrate these Sec24 binding site mutants into the secretome‐GFP library^27^ and imaged the resulting strains using a high content setup (Figure 1A).^28^

Manual examination of the resulting images revealed that A‐ and C‐site mutations altered the localization of only a handful of proteins, the majority of which were known cargo receptors as expected. Four members of the p24 family, Erp1, Erp2, Erp4 and Erv25, were redistributed from the ER to an ER/punctate localization on the background of mutation in the Sec24 C‐site (Figure 1B). Three proteins showed altered localization in both A‐ and C‐site mutants: 2 known cargo receptors, Erv41 and Erd2, and a putative ER protein with unknown function, Ydl121c (Figure 1C). Erd2 is the retrieval receptor of HDEL bearing proteins and Erv41 is a retrieval receptor of Gls1, Fpr2 and other non‐HDEL bearing proteins.^29^, ^30^ We therefore hypothesized that Ydl121c may also be a cargo receptor. Indeed, colocalization studies revealed that on the background of Sec24 mutants, Ydl121c‐GFP moved from an ER localization to being colocalized with the COPI marker, Cop1‐mCherry (Figure S1).

### 2.2 Ydl121c interacts genetically and physically with Pma1

**Figure 2:**
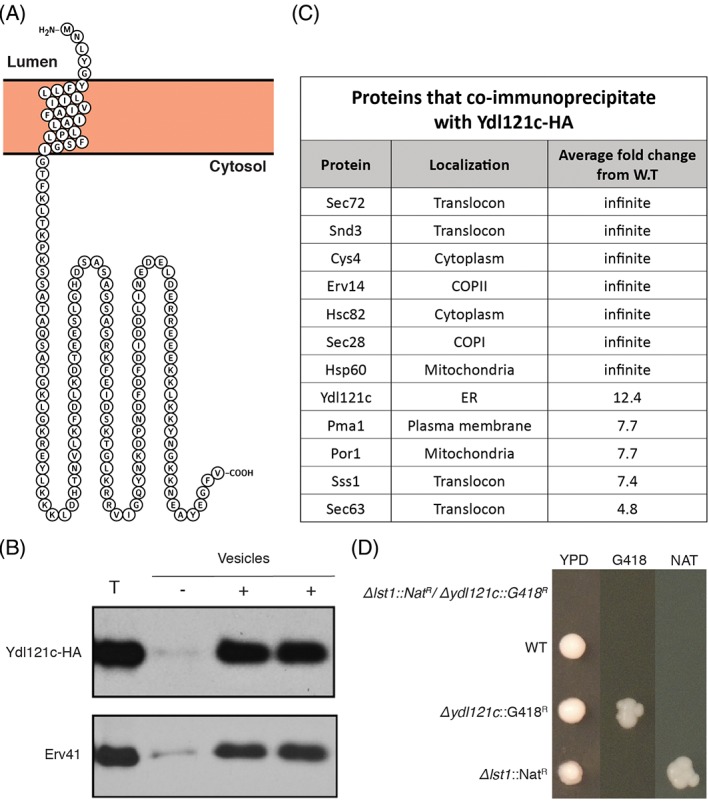
Genetic and physical interactions of Ydl121c suggest Pma1 as a possible cargo protein. A, Schematic of the predicted topology of Ydl121c. B, Ydl121c‐HA is packaged into COPII vesicles. Microsomal membranes were incubated with COPII proteins and GTP (+) or GDP (−), vesicle fractions were separated from total membranes (T) and detected using immunoblotting with αHA/αErv41 antibodies. Erv41 served as a positive control for vesicle formation. C, Affinity precipitation of HA‐tagged Ydl121c followed by mass spectrometry revealed Ydl121c physical interactors and suggests Pma1 as its possible cargo. The table shows all proteins enriched more than 4‐fold in the sample compared with control (WT cells). D, Tetrad analysis of ascospores resulting from meiosis of [*ydl121c*+/− *lst1*+/−] diploids reveals synthetic lethality between the 2 genes. *Δydl121c* strain is G418^R^ and *Δlst1* strain is Nat^R^

Ydl121c is a small, type I, membrane protein of 149 amino acids (aa), harboring a single transmembrane domain (TMD) with only 6 N‐terminal aa facing the ER lumen and a cytosolic domain of 123 aa (Figure 2A).^31^, ^32^ If indeed Ydl121c has a role in ER to Golgi traffic it should bud from the ER in COPII vesicles. To test this we used an in vitro budding assay, which showed that indeed HA‐tagged Ydl121c is actively packaged into COPII vesicles (Figure 2B).

To test if Ydl121c is a cargo receptor we sought to identify potential cargo. We first searched for physically interacting partners by immunoprecipitation of HA‐tagged Ydl121c followed by mass spectrometry. Mass spectrometry identified 11 reproducible, high‐confidence interactors (Figure 2C). Seven of the interactors reside in the endomembrane system, including 4 subunits of the auxiliary ER translocation channel (Sec72, Snd3, Sss1, Sec63)^33^, ^34^ and 1 component each of COPI vesicles (Sec28) and of COPII vesicles (Erv14). While their specific relevance remains to be investigated, these interactors are consistent with the ER residence of Ydl121c and may reflect its cycling in the early secretory pathway. Of the endomembrane system proteins, only Pma1 was an interactor that is not resident in the early secretory pathway. Since Pma1 was enriched as much as Ydl121c itself in the pull‐downs, we considered Pma1 a candidate cargo client for Ydl121c.

In parallel to uncovering its physical interaction partners, we explored the genetic interactions of Δ*ydl121c* using a synthetic lethality screen. We crossed a *Δydl121c* query strain with the yeast haploid deletion and hypomorphic (DAmP) collections.^35^, ^36^ Of particular interest among the small number of synthetic lethal interactors were *Δlst1*, the Sec24 homolog that promotes efficient ER exit of Pma1, and *Δbrp1,* a deletion of a dubious open reading frame that leads to downregulation of *PMA1* 37, 38 (Table S4). To verify these interactions, we performed a manual cross between a strain carrying the *Δydl121c* allele and one carrying a *Δlst1* allele. Following meiosis we used tetrad dissection to isolate progeny. Consistent with the systematic screen results, we could not recover viable spores that bore both *Δydl121* and *Δlst1*, confirming a synthetic lethal interaction between these genes (Figure 2D). The finding that deletion of these 2 genes—a major regulator of Pma1 trafficking and a hypomorphic allele of *PMA1*—causes synthetic lethality with *Δydl121* implies that in cases of perturbed trafficking or synthesis of Pma1, Ydl121c becomes essential. These results support our hypothesis that Pma1 is a client of the candidate cargo receptor Ydl121c.

Pma1 is an abundant and essential yeast plasma membrane protein that functions as a H^+^‐ATPase.^39^ This 100 kDa protein contains 10 TMDs and oligomerizes into dodecamers during its synthesis in the ER.^12^, ^40^ Given its abundance and essentiality, it is perhaps not surprising that Pma1 requires efficient and tightly controlled mechanisms of ER exit and accessory factors to promote its biogenesis and traffic.

### 2.3 Ydl121c is a specific cargo receptor for Pma1

**Figure 3:**
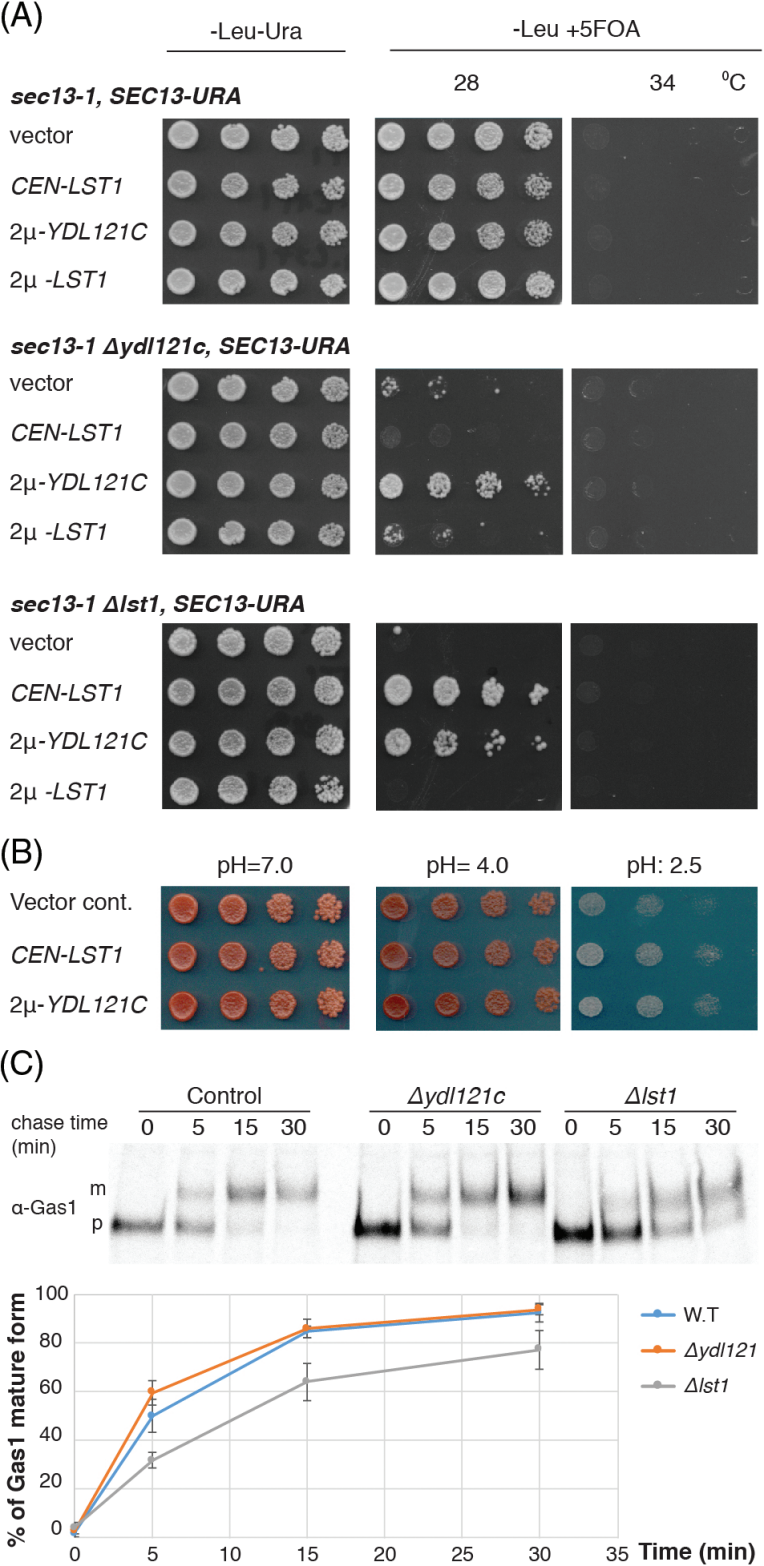
*YDL121C* and *LST1* have parallel roles. A, *YDL121C* and *LST1* show similar genetic interactions with *SEC13*. Strains containing deletions in *ydl121c* or *lst1* on the background of the temperature sensitive mutation *sec13‐1* were spotted as serial dilutions onto media containing 5‐FOA to counter‐select for the *SEC13‐URA3* plasmid and test viability in restrictive temperatures. On standard media (left panels), all strains grew uniformly. In the absence of *SEC13* (5FOA, right panels) *Δydl121c* or *Δlst1* were both synthetic sick. Overexpression (O.E) of *YDL121C* was able to rescue the deletion of *lst1*. Native or overexpression of *LST1* could not rescue the deletion of *ydl121c*. B, *YDL121C* O.E. can rescue *Δlst1* growth delay on low pH. *Δlst1* strains were grown in different pH levels. The known growth delay phenotype of *Δlst1* in low pH was rescued by native expression of LST1 and O.E. of *YDL121C* to the same extent. C, *Δydl121c* does not affect trafficking of Gas1 (another Lst1‐dependent substrate). Pulse‐chase maturation experiment following Gas1 maturation over time demonstrates that *Δlst1* but not *Δydl121c* affects the export rate from the ER (seen by glycosylation states: m‐mature p‐premature) of Gas1 (*n* = 3, mean ± SD)

The Sec24 subunit that affects Pma1 trafficking the most, Lst1 (*l*ethal with *s*ec‐*t*hirteen), was originally identified through its synthetic lethal interaction with the COPII coat component, Sec13. If Ydl121c is similarly required for efficient ER export of Pma1, we reasoned that it might also be lethal with the temperature‐sensitive (ts) *sec13‐1* allele. Indeed when *Δydl121* or *Δlst1* mutations were introduced into the *sec13‐1* strain, they were both synthetic lethal even at the permissive temperature of 28°C (Figure 3A). Moreover, overexpression of *YDL121C* could rescue the lethal phenotype of *sec13‐1/Δlst1*. The fact that *LST1* and *YDL121C* show similar interaction with *sec13‐1* and the capability of *YDL121C* overexpression to overcome *LST1* loss strongly suggest a parallel role for these 2 proteins. In addition, overexpression of *YDL121C* could rescue loss of *lst1* at pH = 2.5, a condition that would require efficient Pma1 export, to the same level as *LST1* itself (Figure 3B). Again showing that Ydl121c can facilitate Pma1 trafficking independently of Lst1 at least to some extent.

We next asked whether Ydl121c is specific to Pma1 or may be required for the packaging of other Lst1‐dependent cargoes, such as GPI‐anchored proteins (GPI‐APs). We therefore examined the maturation of the GPI‐AP, Gas1, using pulse‐chase analysis, where export from the ER and delivery to the Golgi causes a shift in molecular weight from the precursor (p) to the mature (m) form that can be readily quantified. Whereas Gas1 maturation was significantly slowed in a *Δlst1* strain relative to wild‐type, the *Δydl121c* strain showed no such defect (Figure 3C). This suggests that Ydl121c is a specific cargo receptor for Pma1 and does not participate in Lst1‐dependent GPI‐AP trafficking.

### 2.4 Ydl121c and Ykl077w may work together to promote efficient Pma1 maturation

**Figure 4:**
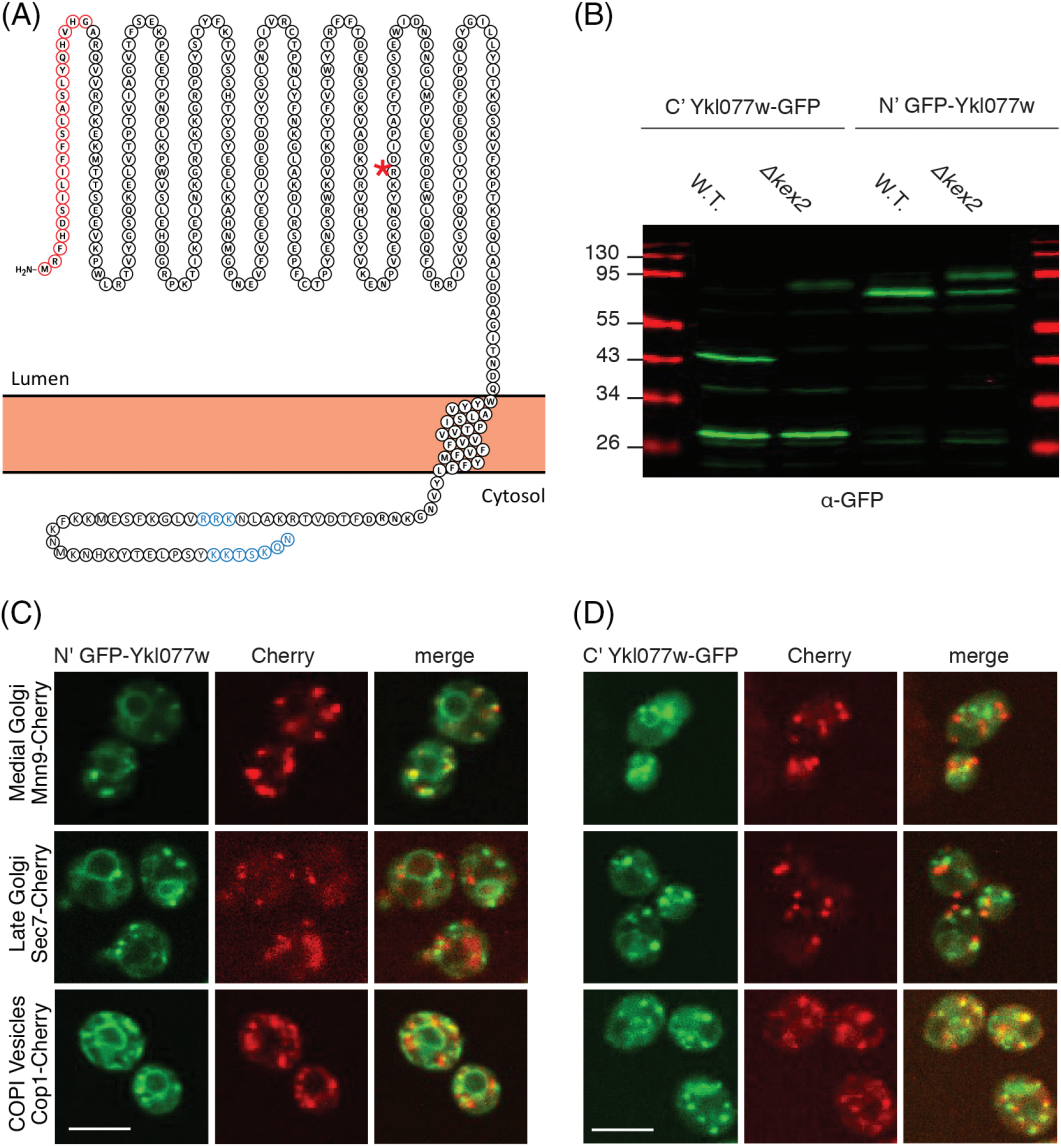
Ykl077w is an uncharacterized binding partner of Ydl121c. A, Schematic of the predicted structure of Ykl077w. Red circles represent the predicted signal peptide, blue circles represent predicted retrieval motifs, red asterisk indicates the Kex2 cleavage site. B, Ykl077w cleavage by Kex2 gives rise to 2 products of proteolytic processing: Ykl077w‐N′ and Ykl077w‐C′. The Kex2‐dependent size‐shift was visualized using western blotting in control (WT) or *Δkex2* strains for either C′ tagged Ykl077w or N′ tagged Ykl077w under control of their native promoter. The full‐length protein is ~64 kD. Ykl077w‐N′ is ~46 kD and Ykl077w‐C′ is ~18 kD. For further details see Figure S2. C, Ykl077w‐N′ colocalized mainly with the mid‐Golgi marker, Mnn9 and is also present in the ER (which could be the unprocessed form). D, Ykl077w‐C′ mainly colocalizes with a COPI marker. Some colocalization was also observed with Golgi markers. Bar = 5 µm

To further characterize Ydl121c function, we searched again for potential interactors, using this time an in vivo protein interaction assay that works by split DiHydro Folate Reductase complementation (Split DHFR screen).^41^ The strongest interaction that we detected was with an uncharacterized protein, Ykl077w. *YKL077W* encodes an open reading frame that should give rise to a 392aa protein with a predicted signal sequence at its N‐terminus and a single TMD near the C‐terminus. The short cytosolic region bears 2 putative retrieval motifs KRR and KKxxKxx (Figure 4A). Interestingly, Ykl077w was previously shown to be cleaved by the Ca^+^ dependent serine protease, Kex2. This cleavage was shown to give rise to a ~30 kDa transmembrane protein^42^ when the C′ portion was followed. However, it was not determined whether the N′ portion also gives rise to a stable fragment or whether it is only necessary for the biogenesis of Ykl077w. To follow the N′ fragment we N′ tagged Ykl077w and found that indeed, the N′ itself also gives rise to a mature protein (Figure 4B). Apparently this phenomenon is common to other Kex2 substrates as well.^43^ The full‐length form of the protein is short lived and can only be detected on the background of *Δkex2*. Interestingly, Ykl077w‐N′ runs at a much higher molecular weight than would be predicted by its length, suggesting that it is highly glycosylated (Figure 4B). In support of this, many sites on Ykl077w‐N′ were recently shown to be *O*‐mannosylated in a high throughput analysis of glycosylation in yeast^44^ (Figure S2). We performed pull downs of Ykl077w‐N′ and Ykl077w‐C′, which demonstrated physical interactions with most of the Golgi glycosylation machinery (Tables S5 and S6). Existing genetic interaction data indeed shows that the glycosylation complex and *ykl077w* have a buffering interaction suggesting a co‐dependence.^45^ Both Ykl077w‐N′ and Ykl077w‐C′ showed significant enrichment for a physical interaction with Ydl121c (Tables S5 and S6). In contrast to Ydl121c, neither Ykl077w N′ nor C′ showed a physical interaction with Pma1.

To see whether the 2 Ykl077w derived proteins function in the same cellular compartment we colocalized GFP‐Ykl077w and Ykl077w‐GFP with a variety of markers for the endomembrane system and found that while the full length or Ykl077w‐N′ is mostly colocalized with the ER and mid‐Golgi (Figure 4C), the Ykl077w‐C′ is mostly colocalized with COPI vesicles although some colocalization was seen with Golgi markers as well (Figure 4D). The fact that each fragment has a different steady‐state localization should be taken into consideration when attempting to understand the role of Ykl077w in sorting or maturation in the early secretory pathway.

### 2.5 Loss of Ykl121c or Ykl077w affects Pma1 maturation

**Figure 5:**
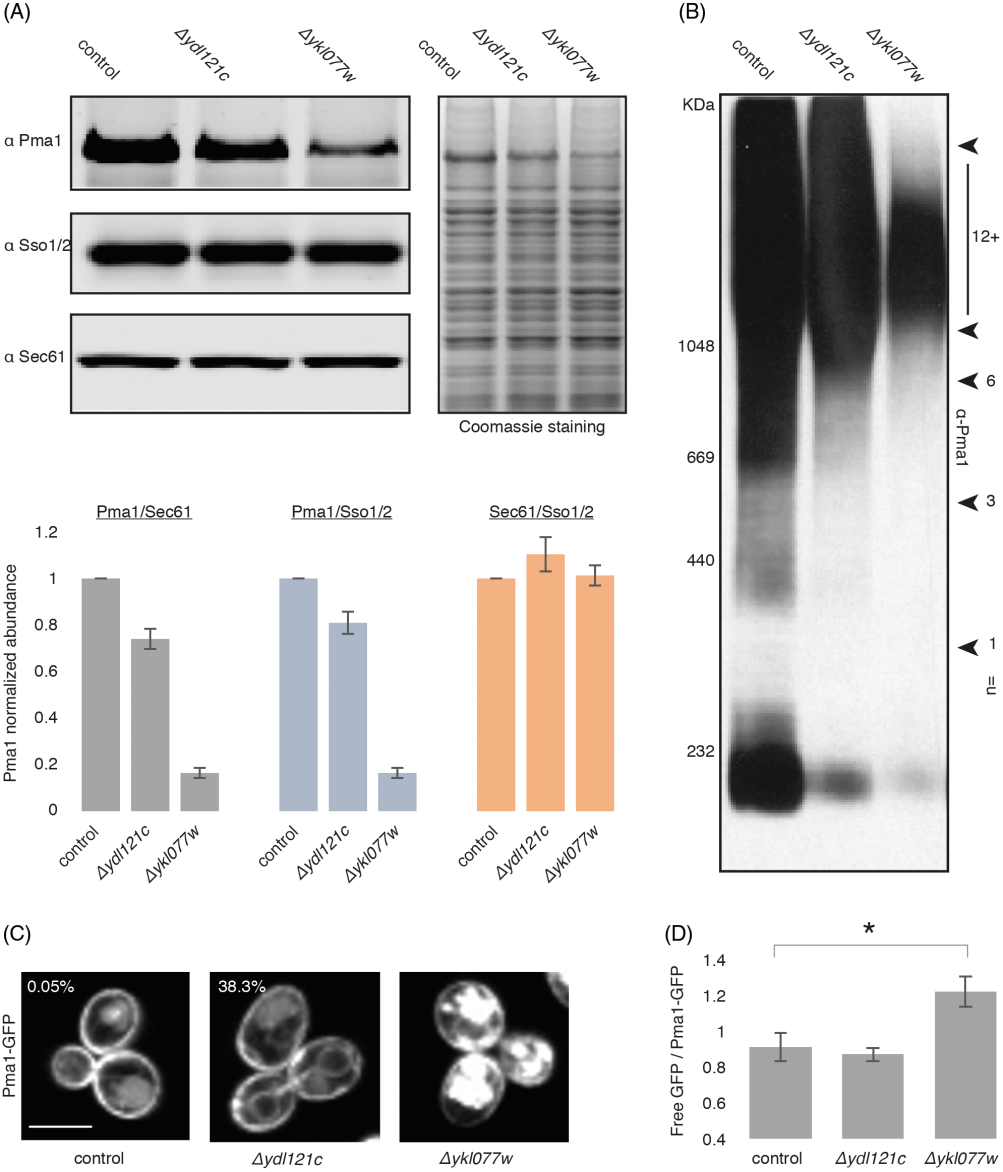
Ydl121c and Ykl077w are essential for optimal Pma1 maturation. A, Left: Protein extraction and separation on SDS‐PAGE shows that Ydl121c and Ykl077w affect Pma1 levels. Right: Comassie staining of total membrane proteins shows no change in general protein abundance. Bottom: Quantification of Pma1 signal relative to 2 other membrane proteins (Sso1/2 or Sec61) shows specific reduction of more than 20% in Pma1 abundance on the background of *Δydl121c* and more than 80% on the background of *Δykl077w* (*n* = 6, mean ± SE). B, Ydl121c and Ykl077w affect the abundance of Pma1 oligomers. Protein extraction and separation on Blue native (BN)‐PAGE show reduction in Pma1 abundance on the background of *Δydl121c* and *Δykl077w* in all oligomeric states. Arowheads indicate different multimeric states of Pma1. Assignment of stoichiometries was done according to References 40, 46. C, Ydl121c and Ykl077w affect Pma1 localization at steady state. After 72 hours of continuous growth in logarithmic phase, maintained by consistent dilution of the culture, Pma1 showed ER retention on the background of *Δydl121c* (quantification shown in upper left corner of images) and much stronger vacuolar staining on the background of *Δykl077w*. D, Quantification of western blot detecting both Pma1‐GFP and free GFP (using anti‐GFP antibodies) on the background of *Δydl121c* and *Δykl077w*. Calculation of free GFP/Pma1‐GFP ratio shows 30% higher abundance of free GFP on the background of *Δykl077w* relative to control strain (*n* = 3, mean ± SE)

To assess the direct influence of Ydl121c and Ykl077w on Pma1 we quantified untagged, endogenous, Pma1 levels. The *Δydl121c* strain showed a 20% reduction in Pma1 abundance relative to the wild‐type (WT) control and the *Δykl077w* strain showed a reduction of more than 80% in Pma1 abundance (Figure 5A).

One key feature of Pma1 biogenesis is its assembly into larger multimers, which occurs during early stages of maturation within the ER.^40^, ^46^ We therefore tested the abundance and assembly of Pma1 under native conditions using blue native gel electrophoresis. In all strains we could readily detect the fully assembled complex as well as several intermediate complexes of Pma1, however, both *Δydl121c* and *Δykl077w* strains showed a reduction of Pma1 abundance in all forms (Figure 5B).

To visualize the cellular compartment in which Pma1 is most affected, we introduced Pma1 C‐terminally tagged with GFP (Pma1‐GFP) on the background of *Δydl121c* or *Δykl077w*. During continuous logarithmic growth, when Pma1‐GFP is normally localized to the plasma membrane and vacuole (Figure 5C), loss of Ydl121c caused an ER retention phenotype and loss of Ykl077w caused accumulation in the vacuole lumen (Figure 5C), which could explain the reduced abundance of Pma1 in this background. Indeed, measurement of free GFP, which is stable in the vacuole lumen^47^ shows that *Δykl077w* accumulates free GFP (Figure 5D). Taken together, our data suggests that Ydl121c and Ykl077w both contribute to Pma1 maturation and sorting albeit in different ways.

## 3 DISCUSSION

Pma1, the yeast H+ − ATPase and the major regulator of cytoplasmic pH and plasma membrane potential, is the most abundant protein in the plasma membrane.^39^ Pma1 maturation is highly complex as it oligomerizes to hexamers and dodecamers within the ER in a manner dependent on ceramide biosynthesis. Its traffic relies on incorporation into ER‐derived vesicles most likely enriched in sterols and sphingolipids, and harboring the Sec24 homolog Lst1.^12^, ^40^ Its further trafficking from the Golgi to the plasma membrane is aided by 2 additional proteins, Ast1 and Ast2, that stabilize it in ceramide‐rich domains in the membrane.^48^, ^49^ While searching for proteins that take part in ER‐to‐Golgi traffic we found 2 novel, uncharacterized proteins that seem to promote and regulate the sorting of Pma1: Ydl121c that has been named Exp1 (for ER eXport of Pma1, Chris Kaiser and Darcy Morse, personal communication) and Ykl077w that we now name Psg1 (Pma1 Stabilization in the Golgi).

**Figure 6:**
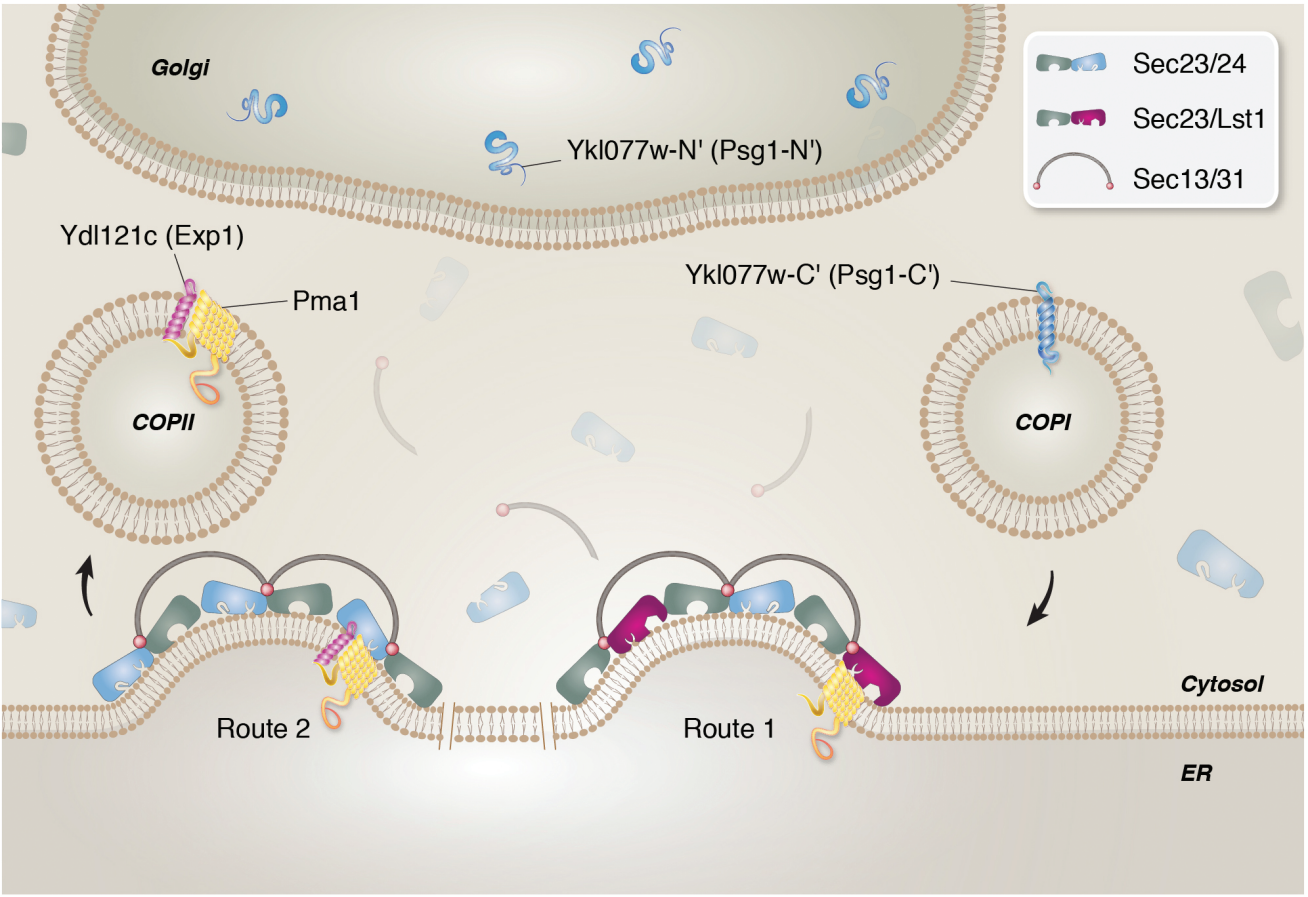
A schematic model of the roles of Ydl121c (Exp1) and Ykl077w (Psg1) in Pma1 sorting and maturation. Based on our observations we suggest that Ydl121c (from hereon called Exp1) is a Pma1 cargo receptor. Because it can promote ER to Golgi traffic independently of Lst1 we conclude that it is not essential for Pma1 trafficking through COPII vesicles constructed of Sec24 and Lst1 isoforms but rather that it is essential in vesicles constructed of Sec24 proteins only. This suggests that there exist 2, parallel, routes to enable Pma1 exit. Ykl077w (from hereon called Psg1) is cleaved giving rise to 2 cleavage products: Ykl077w‐N′ (Psg1‐N′) that is localized mainly to the ER and mid‐Golgi and Ykl077w‐C′ (Psg1‐C′) localized mainly to COPI vesicles. Deletion of the complete gene causes instability of Pma1 and enhanced vacuolar degradation. Considering its localization and deletion phenotype yet lack of physical interaction with Pma1, we suggest that Ykl077w might have an indirect role in retrieval of immature Pma1 from the Golgi back to the ER or in promoting its maturation in the Golgi

Our experiments suggest that Exp1 is a cargo receptor for Pma1 to exit the ER. First, similar to other cargo receptors it cycles between the ER and Golgi and is affected by Sec24 cargo‐binding site mutants. In support of our findings, Exp1 was found to be enriched in COPI/COPII vesicles in 2 independent studies^24^, ^50^ and we found that it is packaged into COPII vesicles in vitro. Second, Exp1 physically binds Pma1 as would be expected for a cargo receptor. Exp1 also binds several ER translocation channel components raising the intriguing possibility that it binds Pma1 soon after translocation and escorts it to exit sites. Third, *Δexp1* shows synthetic lethality with a hypomorphic allele of *PMA1* and with *Δlst1*, the *SEC24* homolog dedicated to Pma1 export from the ER. These genetic interactions are consistent with a functional role in increasing the rate or fidelity of ER export of a specific substrate. Finally, loss of Exp1 causes ER retention of Pma1‐GFP and reduced steady‐state levels of all multimeric forms of the protein. We would therefore hypothesize that Exp1 works to package Pma1 into Sec24 containing vesicles in a manner that is parallel to Lst1 (Figure 6).

The function of Psg1 is less clear. We showed that Psg1, cleaved by the Ca^+^ dependent serine protease Kex2, gives rise to 2 stable cleavage products that we now name Psg1‐N′ and Psg1‐C′ (Figure 6). Our experiments do not differentiate which of the 2 products of Psg1 affect Pma1 trafficking as all of our genetic ablations remove both isoforms. It remains to be determined in the future whether the 2 proteins act together or separately to facilitate Pma1, maturation and if the short‐lived, full‐length, protein form is also active. Moreover, we could not detect a physical interaction between Psg1 and Pma1, suggesting that its mode of action is indirect. However, losing Psg1 caused increased vacuolar accumulation of Pma1‐GFP and dramatically reduced Pma1 levels. This could be due to faulty sorting of the protein to the vacuole instead of to the plasma membrane or as a consequence of increased endocytosis. It may also be that Psg1 has a role in ER retrieval of escaped Pma1 monomers that are immature or misassembled that have escaped the ER to give them another chance of assembly, and in its absence, Golgi quality control mechanisms route the monomer to the vacuole.^51^

Interestingly the N’ tagged Psg1 showed physical interactions with ERAD‐related proteins involved in Pma1 quality control (Eps1, Cdc48, Ubx2, Ssm4) (Table S5)^52^ supporting a role in maturation quality control for this protein. The C′ Psg1 showed physical and genetic interaction with the Tlg1/2 SNARE complex (Tlg1, Tlg2, Vti1) (Table S6) suggesting a role in modulating vesicular trafficking. Psg1 was also found to be associated with Golgi mannosyl transferase complexes and exhibits a genetic profile correlative with being associated with the glycosylation pathway,^45^ hence Psg1 might couple Pma1 sorting and maturation in the early secretory pathway with the glycosylation machinery. In validation of this, we found that deletion of *MNN11*, one of the Golgi mannosyl transferase subunits, caused enhanced vacuolar degradation of Pma1 similar to the deletion of *PSG1*. However the double deletion of *PSG1*, *MNN11* had a suppressor effect and completely abolished the vacuolar degradation phenotype (Figure S3).

Because *Δpsg1* and *Δexp1* did not show a negative genetic interaction with each other but were found to physically interact, we hypothesize that they act sequentially to promote Pma1 sorting between the ER and the Golgi. It can be suggested that Exp1 promotes Pma1 export from the ER to the Golgi while Psg1 has a role in Pma1 maturation or quality control in the Golgi (Figure 6). More generally, our screening strategy allowed us to identify 2 novel players in ER‐Golgi traffic that regulate, one of the most important and abundant proteins in the cell.

## 4 MATERIALS AND METHODS

### 4.1 Yeast strains and strain construction

All yeast strains in this study are based on the BY4741 laboratory strain.^53^ Manipulations were performed using a standard polyethylene glycol/lithium acetate (PEG/LiAC) transformation protocol.^54^ Deletions were verified using primers from within the endogenous open reading frame. Primers for all genetic manipulations were planned by Primers‐4‐Yeast web tool.^55^ All strains and plasmids used in this study are listed (Tables S1 and S2). To construct the Secretome GFP library, we initially chose 565 strains that represent a variety of secretory pathway proteins from the C′‐tagged GFP library^25^ To assemble the library, we hand‐picked all possible secretory pathway proteins. The initial array was visualized and only 374 strains displaying a strong and correctly localized GFP signal were put into the final array (a complete list of selected strains is available in Table S3).

The *sec13‐1* double mutant strains were made by transforming the parental *sec13‐1* strain (RSY2069/LMY282) containing a *SEC13‐URA3* plasmid (pLM246) with a PCR‐amplified deletion cassette with overlap to the *LST1* locus (pAG25^56^). The *sec13‐1 exp1::HPHMX4* double mutant strain was made similarly using a deletion cassette (pAG32^56^) and *EXP1* locus overlap.

### 4.2 Yeast media and growth conditions

Cultures were grown at 30°C in either rich medium (1% Bacto‐yeast extract [BD], 2% Bacto‐peptone [BD] and 2% dextrose [Amresco]) or synthetic medium (0.67% yeast nitrogen base with ammonium sulfate and without aa [CondaPronadisa] and 2% dextrose [Amresco], containing the appropriate supplements for plasmid selection).^57^ When needed as selection markers, G418 (200 µg/mL, Calbiochem) or Nourseothricin (Nat) (200 µg/mL WERNER BioAgents) were added. In cases where G418 was required in a synthetic medium, yeast nitrogen base without ammonium sulfate (CondaPronadisa) was added and supplemented with monosodium glutamate (Sigma) as an alternative nitrogen source.

For dilution assay strains were grown to saturation and 1:10 serial dilutions were plated on agarose media containing 5‐FOA to counterselect for the *SEC13‐URA3* plasmid. Plates were incubated at the indicated temperatures for 2 days before imaging.

### 4.3 Pulse‐chase analysis

Maturation of Gas1 was monitored using pulse‐chase analysis as described in Reference 58.

### 4.4 Protein structure prediction

Prediction of protein architecture was performed using Protter.^32^

### 4.5 Automated yeast library manipulations and high‐throughput microscopy

Automated mating procedures and microscopic screening were performed using an automated microscopy set‐up as previously described,^27^, ^28^ using the RoToR bench‐top colony arrayer (Singer Instruments) and automated inverted fluorescent microscopic ScanR system (Olympus). Images were acquired using a 60× air lens with excitation at 490/20 nm and emission at 535/50 nm (GFP) or excitation at 575/35 nm and emission at 632/60 nm (mCherry/RFP). After acquisition, images were manually reviewed using the ScanR analysis program.

### 4.6 Manual microscopy

Manual microscopy was performed using the VisiScope Confocal Cell Explorer system, composed of a Zeiss Yokogawa spinning‐disk scanning unit (CSU‐W1) coupled with an inverted Olympus IX83 microscope. Images were acquired using a 60× oil lens and captured by a connected PCO‐Edge sCMOS camera, controlled by VisView software, with wavelength of 488 nm (GFP) or 561 nm (mCherry/RFP). Images were transferred to ImageJ for slight contrast and brightness adjustments.

### 4.7 In vitro budding into COPII vesicles

Microsomal membranes were prepared from yeast cells expressing HA‐tagged Ydl121c and used for in vitro vesicle formation as described.^7^ In brief, membranes were washed with urea to remove endogenous COPII proteins, then incubated with purified Sar1, Sec23/Sec24 and Sec13/Sec31 in the presence of GDP (negative control) or GTP plus an ATP regeneration system. Vesicles were separated from donor membranes by medium speed centrifugation (15 000*g*) and the vesicle fraction collected by high speed centrifugation (100 000*g*). A fraction of the total donor membranes (T) and the vesicle fractions were analyzed using immunoblotting.

### 4.8 Affinity purification and mass spectrometry

Yeast cells expressing HA tagged Exp1 at the N′ or C′ or GFP tagged Psg1 N′ or C′ were harvested from mid‐logarithmic growth phase. Cells were snap frozen in liquid nitrogen and ground (CryoMill, Retsch, Germany). Resulting powder was solubilized in 1% digitonin (D1414, SIGMA), 50 mM Tris/Cl, 150 mM NaCl, pH 7.5. Proteins were extracted using Monoclonal Anti‐HA − Agarose antibody produced in mouse (SIGMA)/GFP‐Trap (chromotek) for 1 hour followed by 3 washes with 150 mM NaCl, 50 mM Tris HCl pH 7. Bound proteins were released from the beads by a 30 second acidic treatment (0.2 M Glycine pH 2.5), which was neutralized with 1 M Tris pH 9.4. The eluted proteins were digested with 0.4 µg sequencing grade trypsin for 2 hours, in the presence of 100 μL of 2 M urea, 50 mM Tris HCl pH 7.5, 1 mM DTT Resulting peptides were acidified with Trifluoroacetic acid (TFA) and purified on C18 StageTips. LC‐MS/MS analysis was performed on the EASY‐nLC1000 UHPLC (Thermo Fisher Scientific, MA, USA) coupled to the Q‐Exactive mass spectrometer (Thermo Fisher Scientific, MA, USA). Peptides were loaded onto the column with buffer A (0.5% acetic acid) and separated on a 50 cm PepMap column (75 µm i.d. 2 µm beads; Dionex, Thermo Fisher Scientific, CA, USA) using a 4 hour gradient of 5%‐30% buffer B (80% acetonitrile, 0.5% acetic acid). Interactors were extracted by comparing the protein intensities to a WT (BY4741) control.

### 4.9 Western blot analysis

Yeast proteins were extracted either by NaOH or trichloroacetic acid (TCA) protocol as previously described^59^ and resolved on polyacrylamide gels, transferred to nitrocellulose membranes blots, and probed with primary rabbit/mouse antibody against Pma1 (Abcam, UK, ab4645), HA (Covance, NJ, USA, MMS‐101P) or GFP (Abcam, UK ab290). The membranes were then probed with a secondary goat‐anti‐rabbit/mouse antibody conjugated to IRDye800 or to IRDye680 (LI‐COR Biosciences, NE, USA). Membranes were scanned for infrared signal using the Odyssey Imaging System.

### 4.10 Blue native gel electrophoresis

Yeast microsomes were prepared according to.^60^ In brief, spheroplasts of yeast were lysed by dounce homogenisation (25 strokes) in lysis buffer (0.1 M Sorbitol, 20 mM HEPES pH 7.4, 50 mM potassium acetate, 2 mM ethylenediaminetetraacetic acid [EDTA], 1 mM DTT, 1 mM phenylmethylsulphonyl fluoride [PMSF]) at 4°C. The lysates were centrifuged at 1000*g* and the resulting supernatant at 27 000 g for 10 minutes at 4°C. The crude membrane pellet was re‐suspended in lysis buffer and layered onto a discontinuous sucrose density gradient consisting of 1.2 and 1.5 M sucrose. Following centrifugation at 100 000*g* for 60 minutes at 4°C the membranes at the 1.2‐1.5 M sucrose interface were collected and washed twice in lysis buffer. The membrane pellets were re‐supended in membrane storage buffer (50 mM Nacl, 0.32 M sucrose, 20 mM HEPES pH 7.4, 2 mM EDTA containing protease inhibitors) and the protein concentration determined by a standard Bradford assay.^60^

Microsomes were solubilised in ComplexioLyte 48 buffer (1 mg/mL, Logopharm) for 30 minutes at 4°C.^61^ Solubilized extracts were centrifuged at 100 000*g* for 30 minutes at 4°C and supplemented with glycerol (5%) and Coomassie G‐250 (0.3%) and loaded on a 3.5%‐15% linear native polyacrylamide gel. The BN‐PAGE gel was prepared according to.^62^ The gel buffer contained 25 mM imidazole and 500 mM 6‐aminohexanoic acid. The cathode chamber was first filled with cathode buffer B (50 mM Tricine, 7.5 mM imidazole and 0.02% coomassie) and subsequently replaced by cathode buffer B/10 (containing 0.002% coomassie) after the gel running front had covered a third of the desired distance of electrophoresis. The anode chamber was filled with 25 mM imidazole, pH 7.0. A high molecular weight calibration kit for native electrophoresis from GE Healthcare was used as a standard.^61^, ^62^

## EDITORIAL PROCESS FILE

The Editorial Process File is available in the online version of this article

## Supporting information

Editorial Process

**Table S1** Yeast strains used in this paper.

**Table S2** Plasmids used in this paper.

**Table S3** Strains in the GFP secretome library.

**Table S4** *YDL121C* synthetic lethal interactions. Five most consistent *Δydl121c* synthetic lethal interactors from a whole‐genome synthetic lethality screen. The first 2 are *LST1,* the *SEC24* homolog with a specific role in Pma1 ER‐to‐Golgi traffic and *BRP1* a gene located in the upstream region of *PMA1* whose deletion creates a hypomorphic allele of *PMA1*.

**Table S5** N′ GFP‐Ykl077w physical interactors. Most enriched proteins identified in affinity precipitation of GFP‐Ykl077w followed by mass spectrometry. The table shows all proteins enriched more than 4‐fold in the sample compared with control (cells expressing cytosolic GFP).

**Table S6** C′ Ykl077w‐GFP physical interactors. Most enriched proteins identified in affinity precipitation of Ykl077w‐GFP followed by mass spectrometry. The table shows all proteins enriched more than 4‐fold in the sample compared with the control (cells expressing cytosolic GFP).

**Figure S1**. Ydl121c‐GFP colocalizes with COPI markers on the background of sec24‐A or C mutants. Co‐expression of Ydl121c‐GFP on the background of *sec24* mutations with mCherry tagged markers for COPI, COPII or Golgi (Cop1, Sec13 and Vrg4, respectively) show best colocalization with the COPI marker. Bar = 5 µm.
**Figure S2**. Parameters affecting Ykl077w‐N′/C′ size. A, Residues of Ykl077w shown to be *O*‐mannosylated in a high throughput analysis of glycosylation in yeast^44^. Blue letters indicate the *O*‐mannosylation sites, Orange‐signal peptide, purple‐transmembrane domain and red‐Kex2 cleavage site. B, Calculation of Ykl077w‐N′/C′ sizes as assayed using western blot.
**Figure S3**. Microscopy and western blot analysis of Pma1‐GFP in *Δykl077w* and *Δmnn11* single and double mutants. *Δmnn11* and *Δykl077w* buffer each others enhanced Pma1 degradation phenotype. After 72 hours of continuous growth in logarithmic phase, maintained by consistent dilution of the culture, Pma1 showed strong vacuolar staining on the background of *Δmnn11* or *Δykl077w*. Bar = 5 µm. However, double deletion *Δmnn11*/*Δykl077w* rescued this phenotype. Moreover, the ratio of free GFP/Pma1‐GFP as assayed by western blot was similar to WT in the double deletion strain, suggesting reduced vacoular degradation of Pma1 relative to each single mutant. N = 3 bar = ± SE.
