# Supplementary material for: Two novel effectors of trafficking and maturation of the yeast plasma membrane H+‐ATPase
Source: Traffic. 2017 Aug 16;18(10):672–82. doi: 10.1111/tra.12503 (PMC5607100; doi:10.1111/tra.12503)
Supplement: Supplementary file 1 — Editorial Process [file TRA-18-672-s007.pdf]

Title: **Two novel effectors of trafficking and maturation of the yeast plasma membrane H$^+$-ATPase**

Authors: Yosef Geva, Jonathan Crissman, Eric C. Arakel, Natalia Gómez-Navarro, Silvia G. Chuartzman, Kyle R. Stahmer, Blanche Schwappach, Maya Schuldiner, Elizabeth A. Miller

Article Type: Original Research

Monitoring Editor	Sharon Tooze
Date Submitted	7 December 2016
Date for Decision 1	4 January 2017
Date Resubmitted	3 May 2017
Date for Decision 2	4 May 2017
Accepted	17 July 2017

Decision and Reviews

Dear Maya,

Thank you for submitting your manuscript "Two novel effectors of trafficking and maturation of the yeast plasma membrane H+-ATPase" for consideration for publication in Traffic. I agree with the referees that the work presented in this paper will be of interest to the readers of Traffic. However, the referees have made several recommendations that I agree will strengthen your conclusions. Although the revisions necessary will require you to include additional experiments, I believe that these will be straightforward and I look forward to receiving your revised paper in the near future. To expedite handling when you resubmit please be sure to include a response outlining how you have addressed each of the referees' concerns.

Sincerely,

Sharon

Sharon A. Tooze, Ph.D.
Co-Editor

Referee's Comments to the Authors

Referee: 1

Comments to the Author
This manuscript describes a new putative cargo receptor for the plasma membrane H+-ATPase. Very few cargo receptors have been identified thus far, so the identification of a new cargo receptor is a significant advance worthy of publication in Traffic. As cargo receptors are known to cycle between the Golgi and ER, the groups of Miller and Schuldiner have used a high-content screen in the context of disrupting the cycling of traffic between the ER and Golgi. This screen led to the identification of two interesting proteins, Exp1 and Psg1. Exp1 is a putative cargo receptor for Pma1, while the role of Psg1 is less clear. Nonetheless, Psg1 clearly regulates Pma1 maturation in some way. Additional experiments will be needed prior to publishing this interesting story.

Major experiments
1) Given the suggested roles of Exp1 and Psg1, it is important to show that both of these proteins co-precipitate with Pma1. The IP results should be in one of the main Figures.

2) In Fig. 4B, some of the bands are too light to see. Please replace with a gel where all the described bands can be easily seen.

3) In Fig. 4C, Sec7 is used as a marker for the early Golgi. This is incorrect. It is a marker for the late Golgi.

4) I am a little confused by the model in Fig. 6. Please explain in more detail.
One class of vesicles has Sec24 and the other Sec24 and Lst1. Yet, this is not explained in the legend.

Minor points

1) The reference to Fig. 2C in the first paragraph of the section entitled "Ydl121c interacts genetically and physically with Pma1" should be Fig. 2B. In the following paragraph the reference to Fig. 2B should be Fig. 2C.

2) Please provide more information for the legend to Fig. 2D.

3) In Fig. 3A the suppression is not so obvious. Please replace with a better Figure or tone down the statement.

4) In Fig. 5B, please explain the arrowheads.

Referee: 2

Comments to the Author
In this manuscript Geva et al investigate the cellular function of yeast Ydl121c (Exp1) and Ykl077w (Psg1), two poorly characterized membrane proteins. Through screening approaches and directed experiments, the authors show that Exp1 and Psg1 function in the biogenesis of Pma1, an abundant plasma membrane ATPase. Exp1 is observed to traffic between ER-Golgi compartments and interacts with Pma1 as well as Psg1. Cells that lack Exp1 accumulate Pma1 in the ER whereas cells devoid of Psg1 result in Pma1 degradation. Based on these results and additional genetic experiments the authors suggest that Exp1 serves as an export receptor for Pma1 while Psg1 operates in post-ER maturation of Pma1. The findings generally support their conclusions and characterization of these new proteins should be a valuable resource for the trafficking community. I do have some suggestions to strengthen this work.

1. I would suggest adding some interpretation for why the sec24-A and sec24C mutants cause the observed mislocalization of GFP-cargo. The screen clearly works to identify potential candidates but a few sentences in the results or discussion on why sec24 cargo binding mutants cause a shift from an ER to puncta distribution would be helpful. Are the puncta thought to be cargo aggregates in the ER or cargo trapped in ERES? Or are these post-ER structures as suggested by Fig. 1A?

2. In the legend to Fig. 4 that describes panel 4B, I would explicitly state that the western blot is developed with anti-GFP antibodies.

3. In the Fig. 4 panel C label, I don't think that Sec7 is considered a marker for the early-Golgi? See the publications PMID: 16699524 and PMID: 22344030 for example.

4. Regarding Fig. 5C, it would be preferable to provide a more quantitative assessment of Pma1 ER accumulation in the exp1 deletion strain. This is a major finding of the study. At a minimum, the number of cells that display ER accumulation of Pma1-GFP in a population of exp1 deletion cells compared to the wild type should be included. Ideally, ER and plasma membrane fractions could be resolved from cell lysates to measure the altered distribution of endogenous Pma1 or Pma1-GFP.
-

Author Rebuttal

Point by point response to reviewers suggestions
Reviewer #1
1) Given the suggested roles of Exp1 and Psg1, it is important to show that both of these proteins co-precipitate with Pma1. The IP results should be in one of the main Figures.
-We agree with the reviewer and have now moved the table of IP results of Exp1 to the new Figure 2C. It can be appreciated that Ydl12c itself is 12.4 enriched in the sample compared with the control Pma1 is 7.7 fold enriched. Psg1 does not seem to interact physically with Pma1 and the dramatic effect of its deletion on Pma1 abundance is probably achieved by an indirect mechanism – a fact that we now explicitly discuss in the manuscript.

2) In Fig. 4B, some of the bands are too light to see. Please replace with a gel where all the described bands can be easily seen.
-To improve visualization of all bands we have now repeated the experiment with both tagged forms of Ykl077w expressed from the native promoter. Since now all bands are with similar intensity, it is easier to display the data well. The new data appear in new Figure 4B.

3) In Fig. 4C, Sec7 is used as a marker for the early Golgi. This is incorrect. It is a marker for the late Golgi.
-We thank the reviewer for picking up on our mistake. We have now re-labeled Sec7 as a late Golgi marker and have removed the Chc1 marker form the figure.

4) I am a little confused by the model in Fig. 6. Please explain in more detail.
One class of vesicles has Sec24 and the other Sec24 and Lst1. Yet, this is not explained in the legend.
-We have now slightly changed the graphic of the figure to better demonstrate our model as well as re-worded the legend and the discussion of the model in the manuscript and hope that our model is now conveyed more clearly.

Minor points

1) The reference to Fig. 2C in the first paragraph of the section entitled “Ydl121c interacts genetically and physically with Pma1” should be Fig. 2B. In the following paragraph the reference to Fig. 2B should be Fig. 2C.
-Thank you for pointing this out – we have corrected it

2) Please provide more information for the legend to Fig. 2D.
-We thank the reviewer for pointing this out – indeed this was not clear. We have now better labeled the figure itself with information on which selection is associated with each gene and have also clarified the legend and hope that the figure is now clearer.

3) In Fig. 3A the suppression is not so obvious. Please replace with a better Figure or tone down the statement.
-We thank the reviewer for pointing this out .We have now chosen a more representative figure for the sec13-1 Δlst1 strain that clearly shows rescue by O.E of YDL121c. In the course of doing this we have also realized that the data is difficult to interpret without the control panel of sec13-1 without an additional mutant background and have added this. Most importantly, we have noticed that our temperature assignments were shifted and have now corrected this. The corrected and controlled panel is now much easier to understand.

4) In Fig. 5B, please explain the arrowheads.
-The arrowheads denote different oligomeric complexes of Pma1. Different analyses of Pma1 assembly agree that such assembly intermediates can be visualized in the gel system used but the exact assignment of stoichiometries differs between authors. Since we did not perform additional experiments to assign stoichiometries yet agree that denoting values on the arrows would make the figures clearer we have now added the assignments as verified before (Lee et.al JBC,2002; Eraso et al Traffic,2010; Witting et.al Nat. Protoc.,2006).

Referee: 2
1. I would suggest adding some interpretation for why the sec24-A and sec24C mutants cause the observed mislocalization of GFP-cargo. The screen clearly works to identify potential candidates but a few sentences in the results or discussion on why sec24 cargo binding mutants cause a shift from an ER to puncta distribution would be helpful.
-We have now added a short discussion on the logic behind the screen in the introduction. Namely, since all known cargo of sites A and C are machinery proteins, mutations in these sites should halt machinery recycling without being lethal. In addition, we have added a reference showing that a mutation in the A site prevents COPII vesicle-fusion to the Golgi.

2….Are the puncta thought to be cargo aggregates in the ER or cargo trapped in ERES? Or are these post-ER structures as suggested by Fig. 1A?
-We thank the reviewer for this excellent question. Our co-localization with three different post-ER markers suggest that these are post-ER structures. To address this we now discuss this issue in the text and have added co-localization with a COPI marker to Supplementary Figure S1.

2. In the legend to Fig. 4 that describes panel 4B, I would explicitly state that the western blot is developed with anti-GFP antibodies.
-We have now added this information into the figure body itself as well as the legend and it indeed clarifies the point – thank you.

3. In the Fig. 4 panel C label, I don't think that Sec7 is considered a marker for the early-Golgi? See the publications PMID: 16699524 and PMID: 22344030 for example.
-Thank you for noticing this mistake. Indeed, as also answered above to Reviewer 1, we now changed the label of Sec7 to a late Golgi marker.

4. Regarding Fig. 5C, it would be preferable to provide a more quantitative assessment of Pma1 ER accumulation in the exp1 deletion strain. This is a major finding of the study. At a minimum, the number of cells that display ER accumulation of Pma1-GFP in a population of exp1 deletion cells compared to the wild type should be included. Ideally, ER and plasma membrane fractions could be resolved from cell lysates to measure the altered distribution of endogenous Pma1 or Pma1-GFP.
-We like this suggestion very much and have now quantified the number of cells that show ER retention in both the WT and Δydl121c strains (the Δykl077w could not be quantified since the vacuolar signal prohibits visualizing ER retention even if it is present). The numbers are now added directly into the figure (Figure 5C) and show that clear ER pattern of Pma1-GFP could be observed in only 0.05% of WT cells compared to 38.3% on the background of Δydl121c

Decision and Reviews

Dear Dr. Schuldiner,

Sharon Tooze asked me to write to you on her behalf to thank you for making the changes in your manuscript "Two novel effectors of trafficking and maturation of the yeast plasma membrane H+-ATPase". This manuscript is now accepted for publication in Traffic.

Thank you for sending this work to Traffic.

Best wishes,
Lisa Hannan
