# Supplementary material for: Two novel effectors of trafficking and maturation of the yeast plasma membrane H+‐ATPase
Source: Traffic. 2017 Aug 16;18(10):672–82. doi: 10.1111/tra.12503 (PMC5607100; doi:10.1111/tra.12503)
Supplement: Supplementary file 8 — Figure S1. Ydl121c‐GFP colocalizes with COPI markers on the background of sec24‐A or C mutants. Co‐expression of Ydl121c‐GFP on the background of sec24 mutations with mCherry tagged markers for COPI, COPII or Golgi (Cop1, Sec13 and Vrg4, respectively) show best colocalization with the COPI marker. Bar = 5 µm. Figure S2. Parameters affecting Ykl077w‐N′/C′ size. A, Residues of Ykl077w shown to be O‐mannosylated in a high throughput analysis of glycosylation in yeast44. Blue letters indicate the O‐mannosylation sites, Orange‐signal peptide, purple‐transmembrane domain and red‐Kex2 cleavage site. B, Calculation of Ykl077w‐N′/C′ sizes as assayed using western blot. Figure S3. Microscopy and western blot analysis of Pma1‐GFP in Δykl077w and Δmnn11 single and double mutants. Δmnn11 and Δykl077w buffer each others enhanced Pma1 degradation phenotype. After 72 hours of continuous growth in logarithmic phase, maintained by consistent dilution of the culture, Pma1 showed strong vacuolar staining on the background of Δmnn11 or Δykl077w. Bar = 5 µm. However, double deletion Δmnn11/Δykl077w rescued this phenotype. Moreover, the ratio of free GFP/Pma1‐GFP as assayed by western blot was similar to WT in the double deletion strain, suggesting reduced vacoular degradation of Pma1 relative to each single mutant. N = 3 bar = ± SE. [file TRA-18-672-s006.docx]

**Two novel effectors of trafficking and maturation of the yeast plasma membrane H^+^-ATPase**

Yosef Geva, Jonathan Crissman, Eric C. Arakel, Natalia Gómez-Navarro, Silvia G. Chuartzman, Kyle R. Stahmer, [Blanche Schwappach](http://biochemie.uni-goettingen.de/index.php?id=305), Maya Schuldiner, Elizabeth A. Miller

1. Department of Molecular Genetics, Weizmann Institute of Science, Rehovot 7610001, Israel.

2. Department of Biological Sciences, Columbia University, New York 10027, USA.

3. Department of Molecular Biology, Universitätsmedizin Göttingen, Humboldtallee 23, Göttingen 37073, Germany.

4. MRC Laboratory of Molecular Biology, Francis Crick Avenue, Cambridge CB2 0QH, UK

Correspondence:

[emiller@mrc-lmb.cam.ac.uk](mailto:emiller@mrc-lmb.cam.ac.uk); [maya.schuldiner@weizmann.ac.il](mailto:maya.schuldiner@weizmann.ac.il)

Supplemental Material

**Table S1: Yeast strains used in this paper**

**Table S2:** **Plasmids used in this paper**

**Table S3: Strains in the GFP secretome library**

**Table S4*: YDL121C* synthetic lethal interactions**

Five most consistent *∆ydl121c* synthetic lethal interactors from a whole-genome synthetic lethality screen. The first two are *LST1,* the *SEC24* homolog with a specific role in Pma1 ER-to-Golgi traffic and *BRP1* a gene located in the upstream region of *PMA1* whose deletion creates a hypomorphic allele of *PMA1*.

**Table S5: N’ GFP-Ykl077w physical interactors**

Most enriched proteins identified in affinity precipitation of GFP-Ykl077w followed by mass spectrometry. The table shows all proteins enriched more than four fold in the sample compared to control (Cells expressing cytosolic GFP).

**Table S6: C’ Ykl077w-GFP physical interactors**

Most enriched proteins identifeied in affinity precipitation of Ykl077w-GFP followed by mass spectrometry. The table shows all proteins enriched more than four fold in the sample compared to the control (Cells expressing cytosolic GFP).

**
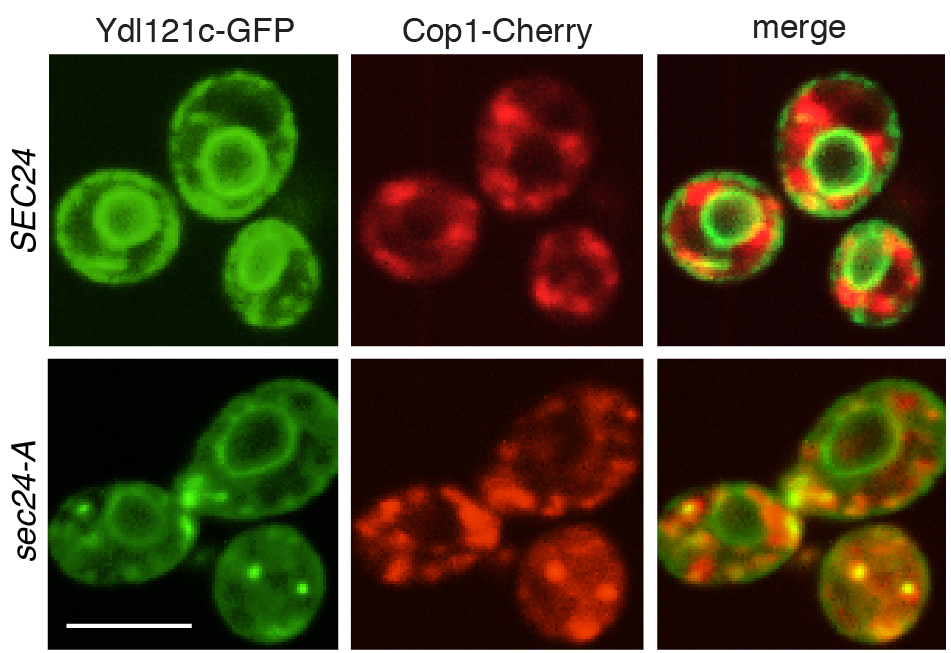
**

**Figure S1: Ydl121c-GFP co-localizes with COPI markers on the background of sec24-A or C mutants.**

Co-expression of Ydl121c-GFP on the background of *sec24* mutations with mCherry tagged markers for COPI, COPII or Golgi (Cop1, Sec13 and Vrg4 respectivly) show best co-localization with the COPI marker. Bar=5μ.

**
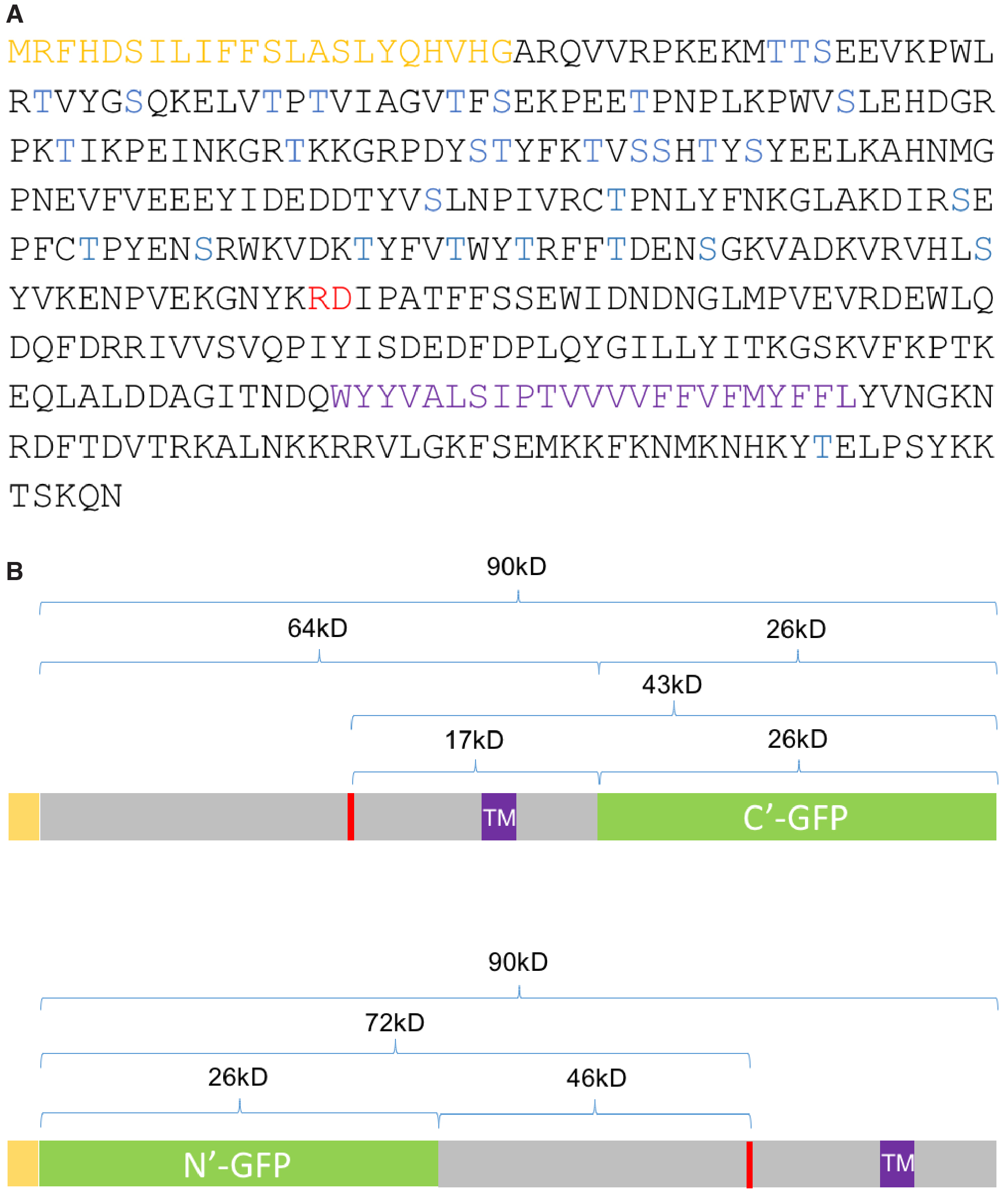
**

**Figure S2: Parameters affecting Ykl077w-N’/C’ size**

(A) Residues of Ykl077w shown to be O-mannosylated in a high throughput analysis of glycosylation in yeast ^44^. Blue letters indicate the O-mannosylation sites, Orange- signal peptide, purple- transmembrane domain and red- Kex2 cleavage site. (B) Calculation of Ykl077w-N’/C’ sizes as assayed by Western blot.

**
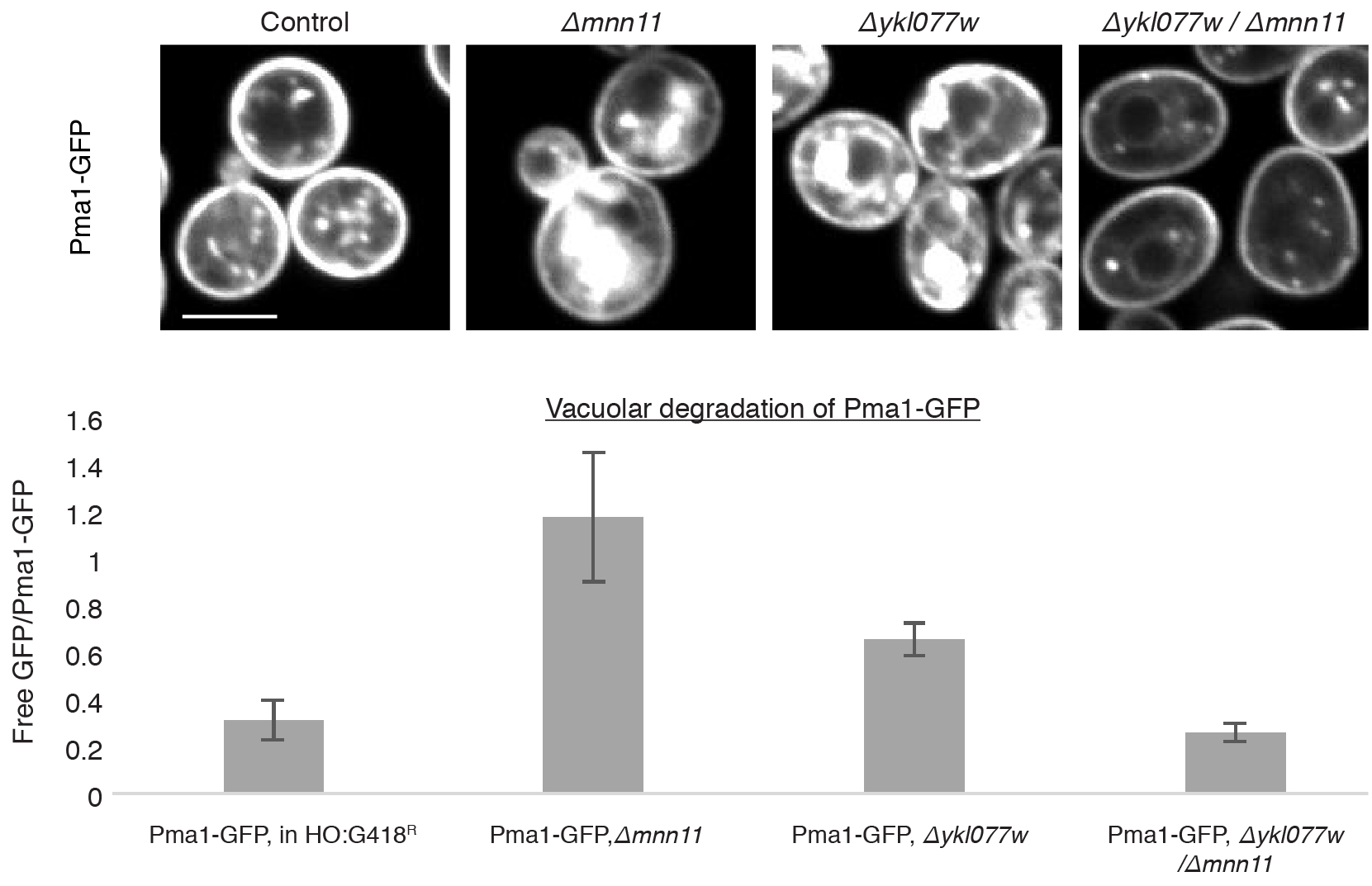
**

**Figure S3: Microscopy and western blot analysis of Pma1-GFP in *Δykl077w* and *Δmnn11* single and double mutants**.

*∆mnn11* and *∆ykl077w* buffer each others enhanced Pma1 degradation phenotype. After 72 hours of continuous growth in logarithmic phase, maintained by consistent dilution of the culture, Pma1 showed strong vacuolar staining on the background of *Δmnn11* or *∆ykl077w*. Bar=5μ However, double deletion *Δmnn11*/*∆ykl077w* rescued this phenotype. Moreover, the ratio of free GFP/Pma1-GFP as assayed by Western blot was similar to WT in the double deletion strain, suggesting reduced vacoular degradation of Pma1 relative to each single mutant. N=3 bar=±SE.
